# A hybrid approach to investigating major management factors for effective highway preventive maintenance

**DOI:** 10.1038/s41598-024-76692-4

**Published:** 2024-10-26

**Authors:** Na Zhao, Yijuan Liu, Huihua Chen

**Affiliations:** 1 School of Traffic and Transportation Engineering, Changsha University of Science & Technology, Changsha, 410114 China; 2 Key Laboratory of Highway Engineering of Ministry of Education, Changsha University of Science & Technology, Changsha, 410114 China; 3 School of Civil Engineering, Central South University, Changsha, 410083 China

**Keywords:** HPM, EFA-SNA-SD method, Management factors, Influence mechanism, Sustainable transportation system, Civil engineering, Energy infrastructure

## Abstract

To resolve the problem of the low management capacity of highway preventive maintenance (HPM), this paper identified and evaluated the major HPM management factors to improve management effectiveness and achieve sustainable highway development. The study conducted a literature review and exploratory factor analysis (EFA) to identify the major HPM management factors. Social network analysis (SNA) was used to distinguish the degree of importance of these factors. A system dynamics (SD) model was developed to explore their patterns of influence. The research identified six dimensions of HPM management, including the management system, management resources, management cognition, management decisions, management technology, and external conditions, along with 26 major management factors. Moreover, information acquisition, system perfection, etc., are key factors; system execution, manager capability, etc., are hub factors; and route selection, machinery allocation, etc., are non-key factors. These factors have a positive impact on HPM management, leading to an upward trend in management effectiveness. The main innovation provided a hybrid and comprehensive approach to identify and evaluate the major management factors for effective HPM. This study can guide managers in developing effective HPM plans, allocating resources more efficiently, improving the overall quality of highway maintenance and forming a sustainable transportation system.

**Supplementary Information:**

The online version contains supplementary material available at 10.1038/s41598-024-76692-4.

## Introduction

Highways play a critical role in modern transportation infrastructure, providing an essential capacity and quality level that fosters sustainable development across the economy, society, humanities, and environment^1,2^. As one of the most crucial infrastructure types, enhancing their sustainability is a top priority to achieve better transportation functions, reduce environmental impacts, ensure passengers’ safety and comfort, and extend highways’ service life, thus creating a better person‒environment–infrastructure relationship in the urban transportation system^3–6^. However, owing to the extensive use of expressways and their prolonged operation time, road damage and deterioration are often unavoidable, leading to a decline in traffic service quality and even jeopardizing personal safety in severe cases^7,8^. Moreover, heavy traffic loads, rising user expectations, and insufficient maintenance funds impose enormous maintenance pressure on highway management^9,10^. Therefore, highway maintenance specifications recommend implementing a prevention-based maintenance policy^3,4,11,12^. According to the Federal Highway Administration, every dollar of preventive maintenance saves $6-$14 in rehabilitation costs. With this type of maintenance, the life of a highway can be extended by 4–10 years, and resources can be conserved^3,4^. In some harsher climates, preventive maintenance is implemented at a rate of 50% or more per year^3^. For example, tens of thousands of kilometers of highway are covered by preventive maintenance each year in the United States. Preventive maintenance covers 20-30% of the total road area^9^. In addition, preventive maintenance reduces carbon emissions from road maintenance by 5-10% and reduces accidents caused by road damage, with accident rates decreasing by 5-20%^7–9^.

Highway preventive maintenance (HPM) involves the implementation of maintenance measures when there are no diseases present or at the initial stage of disease occurrence to prevent the aggravation of problems^4^. This process includes conducting regular inspections and assessments of the highway to detect signs of wear and tear, such as cracks or potholes. Upon identification of such issues, maintenance measures such as sealing cracks or patching potholes can be promptly implemented to prevent further damage^2,13^. An effective HPM is a reasonable approach for sustainable highways, as it prioritizes preventative maintenance over corrective maintenance through early treatment, proactive maintenance, and advanced maintenance^3,4,8,14^. By conducting regular maintenance and repairs, HPM can address minor issues before they become major problems, preventing the need for more extensive and environmentally damaging repairs or reconstruction, which may have significant environmental impacts^5,11,15^, including increased carbon emissions from heavy equipment, construction waste generated from construction activities, and disruption to ecosystems^16^. Moreover, HPM can also improve the energy efficiency of pavement infrastructure by improving the smoothness and ride quality of highways, which results in decreased fuel consumption and greenhouse gas emissions from vehicles. Additionally, some HPM techniques, such as pavement preservation, can use ecofriendly materials and processes that are less harmful to the environment. HPM also identifies and resolves safety hazards through regular maintenance, which improves the safety and accessibility of highways^10,17^. In conclusion, the implementation of an effective HPM can contribute to the cleaner production of highway infrastructures and the sustainability of urban transportation systems^3,4^. However, despite the potential benefits, the widespread adoption of HPM management has faced numerous challenges. These challenges include limited funding, lack of expertise, weak awareness of maintenance management, deteriorating road conditions, lack of political support, and other related management factors^5,9,10,17^.

Effective HPM management requires a combination of technical expertise, financial resources, political support, and effective stakeholder engagement^18–23^. Since these factors are reported in the literature in a piecemeal fashion, a comprehensive understanding of such factors and their dynamic impact from a systems perspective is highly desirable. Therefore, in this study, the aim is to explore the major HPM management factors, their degree of importance, and their patterns of influence through a hybrid approach that combines exploratory factor analysis (EFA), social network analysis (SNA) and system dynamics (SD). Three key questions about HPM management are expected to be answered in this study:

1. What management factors affect the effective HPM?
2. How can the importance degrees of the major HPM management factors be distinguished?
3. How do the major management factors influence the effective HPM dynamically?

The key innovation of this paper lies in its comprehensive and systematic approach in identifying and evaluating the major management factors for effective HPM in the context of the people–environment–infrastructure relationship. By identifying the major management factors and modeling their dynamic interplay, managers can develop more effective HPM plans, allocate resources more efficiently, and ultimately improve the overall quality of highway maintenance. Furthermore, this study can lead to the development of practical tools and techniques for highway maintenance management, which can result in cost savings, improved safety, reduced traffic disruptions, etc. Overall, this can help reduce the negative environmental impacts of transportation infrastructure by prolonging the life of existing infrastructure, reducing the need for costly and resource-intensive repairs and reconstruction, and improving the energy efficiency of pavement infrastructure. This study can provide guidance for improving effective HPM management and promoting an understanding of sustainable infrastructure management, which is critical to the long-term health and sustainability of transportation systems.

This paper is organized as follows: Sect. 2 describes the research methodology, followed by Sect. 3, which identifies the major HPM management factors. Section 4 distinguishes the importance degree of the major HPM management factors. Section 5 explores the pattern of influence of the major HPM management factors using SD. Section 6 presents the implications, limitations and recommendations. Finally, the concluding section summarizes the main contributions of this study.

## Methodology

A hybrid EFA-SNA-SD approach is adopted to screen the major management factors, distinguish their importance degree, and systematically analyze their patterns of influence. EFA is a method used to process multivariate observed variables and perform dimensionality reduction, which allows combining a set of interrelated observed variables into more significant latent variables^24^. It is usually used to determine the number of hypothetical potential variables, structures, dimensions or factors^25^. Thus, EFA can integrate the intricacies of the major HPM management factors in this study. SNA is a methodology that uses mathematical and statistical techniques to analyze social networks and relationships between individuals, groups, or organizations. It helps in identifying patterns and structures within a network and to understand how relationships shape and influence these factors^26^. Therefore, the degree of importance of the major HPM management factors can be reasonably distinguished into key factors, hub factors and non-key factors^27^. SD is a theory that studies the overall behavior of a socioeconomic system by analyzing the feedback structure relationships between the variables within the system. The SD method systematically analyzes the patterns of influence of factors, which is one of the most common procedures used to determine complex systems^28^. Consequently, the SD method is suitable for dynamically exploring the pattern of influence of major HPM management factors.

**Figure 1:**
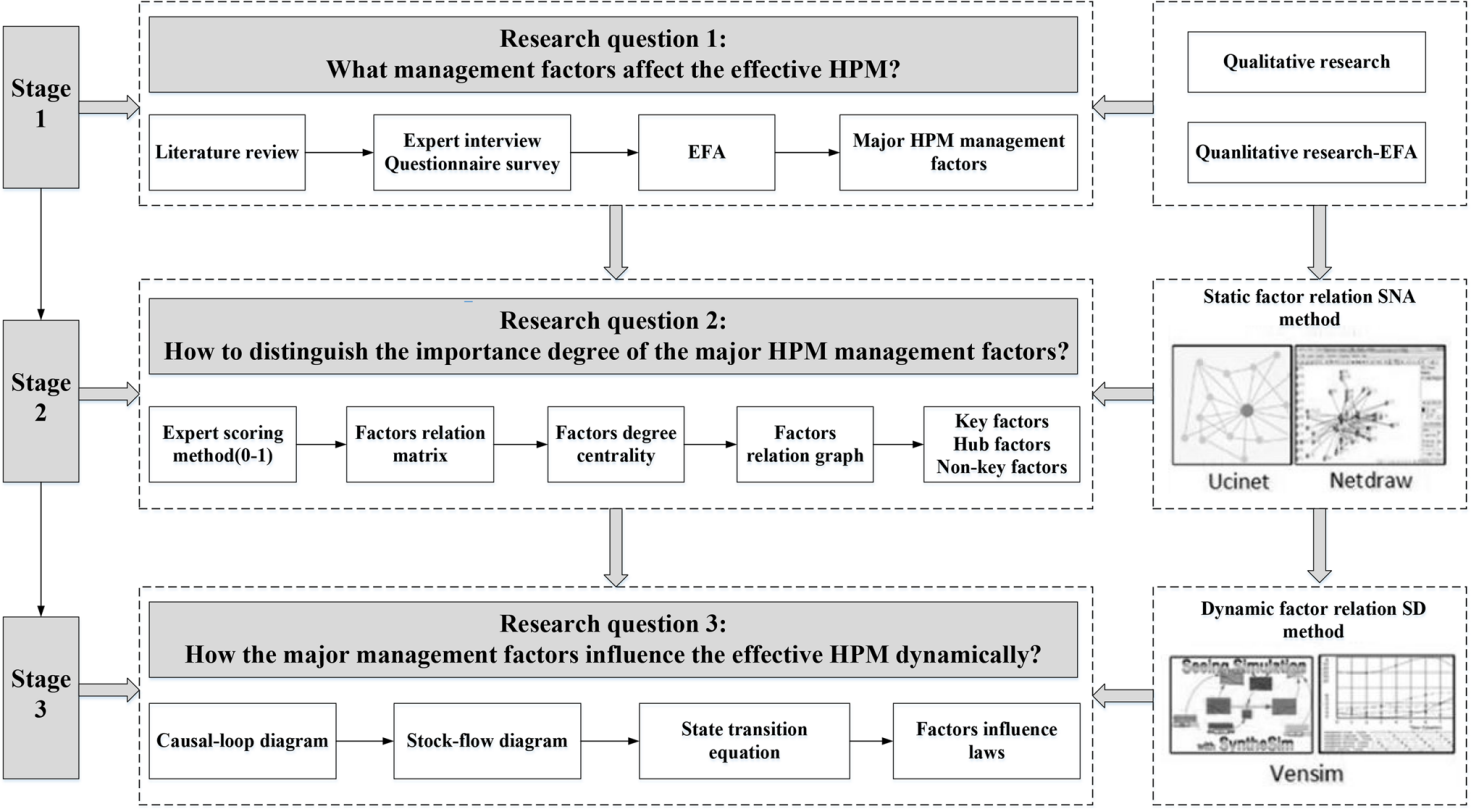
Research route.

As shown in Fig. 1, this research is divided into the following three stages.

- Stage 1: Related literature concerning HPM management is reviewed to preliminarily identify the major management factors in HPM. Expert review, questionnaire surveys and EFA are applied to further identify major management factors in HPM.
- Stage 2: The SNA method is adopted to distinguish the importance degrees of the identified management factors. The relationship matrix of factors is developed on the basis of expert scoring and imported into UCINET 6 software to calculate the relative degree of centrality. The relationship diagram is also drawn with Netdraw software.
- Stage 3: The SD model is used to explore the dynamic patterns of influence among the major HPM management factors. A causal loop diagram is constructed for qualitative analysis, and a stock-flow diagram is constructed for quantitative analysis. The state transition equation is constructed. On the basis of the above steps, simulation analysis is carried out.

## Identification of major HPM management factors

### Literature review

#### Management system

The establishment of an intelligent highway maintenance system is the way to achieve high-quality development of HPM in the new era^29^. It efficiently integrates resources, enhances maintenance efficiency, and ensures highway transportation safety^29–32^. After thorough research, four critical aspects of this system have been identified: system establishment, system perfection, system implementation, and system feedback. First, establishing an intelligent highway maintenance system is essential for improving the maintenance management efficiency, which can help highway management obtain the status information of roads, bridges, tunnels and other facilities in time^29,31,33,34^. Further, the management system needs to be improved to better serve the actual needs of highway maintenance work, which has a high frequency and long period^29,31,34,35^. Furthermore, the management system should be implemented and closely integrated with work practices to ensure smooth implementation^29,33,35,36^. Finally, the implementation effect of the management system is continuously optimized according to the feedback until the system can better adapt to the actual work needs^29,35,36^. This enables precise maintenance and promotes sustainable development.

#### Management resources

The construction and maintenance of large-scale transportation infrastructure require significant resources, emphasizing reasonable and efficient resource allocation^37^. This includes the integrated planning and allocation of machinery, materials, and capital. Among them, machinery is an important tool for highway preventive maintenance^4^. The configuration of machinery should be considered to ensure the performance and quality of machinery to adapt to maintenance needs and ensure the efficiency of machinery, thus improving the effectiveness and economic efficiency of maintenance management^4,7,22,38,39^. In terms of materials, an adequate and reliable material supply and standard material quality can reduce maintenance costs, improve the maintenance effect and ensure the safety, smoothness and sustainability of highways^40–42^. In terms of capital, capital investments and highway revenues are the sources of HPM funding^18^. If there is a shortage of funds, the needs of HPM cannot be met, and maintenance cannot be successfully promoted. Therefore, securing a source of maintenance funding is critical to HPM^1,18,20,43^.

#### Management cognition

In recent years, the growth in traffic trips and the popularization of transportation have led to increased importance in the construction and maintenance of highways. However, the current problems in HPM management are becoming increasingly noticeable. Among them, public awareness of highway maintenance management directly affects the difficulty of HPM management^44–46^. Therefore, it is essential to adopt the “build and maintain” policy from an ideological level and enhance public awareness and participation in maintenance management. Additionally, the degree of personal cognition directly affects the ability of individuals to drive safely and civilly on highways. Raising maintenance awareness and the degree of personal awareness can help reduce the burden of HPM management^47,48^. The department, as the management and implementation unit, can only accurately grasp and implement conservation measures and improve the effectiveness of HPM management if it has an in-depth understanding of the purpose and significance of HPM^49–51^. It is necessary to improve the ability of maintenance managers and the sense of responsibility of maintenance staff to improve the quality and efficiency of maintenance work^41,52–54^. Only in this way can the smooth implementation of HPM management can be guaranteed, and a more convenient and safe highway travel environment, be provided for society and the public to achieve sustainable development.

#### Management decision

The heavy workload of HPM tasks and the inadequate maintenance management decision-making system urgently require the development of a scientific and efficient management mode that can meet the demand for quick, efficient, and high-quality decision-making under high-load maintenance tasks^3,30,43^. The core issue of HPM is “the right maintenance measures at the right time on the right section of road to achieve the best maintenance benefits”^55^. Considering traffic flow, vehicle type, road conditions and other factors, maintenance managers scientifically and reasonably select routes and develop optimal maintenance time nodes to minimize safety risks during highway operation^56–60^. Additionally, when maintenance measures are carried out, their effectiveness needs to be measured to predict the benefits of maintenance work^14,16,43^. This allows adjustments to the maintenance measures to achieve optimal maintenance results. In addition, the costs of maintenance work are a factor that should not be overlooked^12,15,61^. Maintenance costs are directly related to the development, implementation and effectiveness of management decisions. Reasonable control of maintenance costs is conducive to realizing the economic benefits of maintenance work^11^. Overall, management decisions improve the scientific maintenance methods for the HPM management department to ensure scientific construction maintenance and efficient operation management of highways^12,62^.

#### Management technology

With modern maintenance technology and efficient management, highways can operate normally and continuously improve their service level. To address heavy maintenance tasks, new technologies, materials, and techniques can be utilized to increase pavement durability and extend the highway’s service life^11,23,56,63–66^. However, the technical level of existing maintenance personnel is inadequate^67^. Therefore, it is especially important to improve the personal technical level, which will help improve the effectiveness of maintenance management^22,68,69^. Moreover, highway maintenance information is critical. Information acquisition and utilization can help HPM managers gain a comprehensive understanding of the conditions and problems of highways so that effective maintenance programs can be developed^70–73^. This helps maintenance managers control maintenance costs and improve resource utilization. Advanced data analysis techniques can be employed to analyze and extract relevant information from highway maintenance data, guiding the development of maintenance work^19,63,64^.

#### External conditions

The success of HPM is dependent not only on internal management mechanisms but also on external factors. Government attention can increase the investment and support of HPM management, promote the innovation and development of HPM management technology, and provide support to ensure the safety and smooth flow of highways^18,21,74,75^. Market competition helps the development of the highway maintenance industry, improves service quality and reduces maintenance costs^76,77^. Moreover, the risks of maintenance work should receive sufficient attention, and appropriate precautions should be taken to minimize potential impacts^78,79^. In addition, maintenance work is often carried out in fields, open air and other environments that are vulnerable to climatic factors^15,56,80,81^. Therefore, it is important to consider the external factors of HPM management comprehensively to increase management efficiency and improve the quality of maintenance work. This is critical to ensure the safety and smoothness of highways.

On the basis of the literature review, it was found to be necessary to study the major HPM management factors and screen the major HPM management factors, as shown in Table 1.

**Table 1:** Summary of HPM management factors and relevant literature references.

Dimension	Factor	Literature source	Point
Management system	System establishment	Elassy, et al.^29^, Defu Che and Zhao^31^, Xu Qiao, et al.^33^, Yun Hou, et al.^34^	The establishment of the management system can help the highway management to obtain timely information on the status of the road, bridges, tunnels and other facilities.
	System perfection	Elassy, et al.^29^, Defu Che and Zhao^31^, Yun Hou, et al. ^34^	According to the characteristics of HPM, it is essential to perfect the management system to make it better serve the actual needs.
	System execution	Elassy, et al.^29^, Xu Qiao, et al.^33^, Shaojin Zhang, et al.^35^, Mingming Zhou and Zhou^36^	To closely integrate work practices and ensure the successful implementation of maintenance work, the management system needs to be executed.
	System feedback	Elassy, et al.^29^, Shaojin Zhang, et al.^35^, Mingming Zhou and Zhou^36^	To ensure the effectiveness of the implementation of the management system, it is essential to continuously optimize the management system based on feedback.
Management resource	Machinery allocation	Zhu ^7^, Lv, et al.^38^, Mirheli, et al.^39^	Properly allocated machinery can ensure mechanical quality and performance.
	Material supply	Chen, et al.^4^, Mohamed and Tran^40^, Liu, et al.^37^	Ensure adequate material supply to prepare for maintenance work.
	Material quality	Chen, et al.^4^, Mohamed and Tran^40^, JingHai He, et al.^42^	Material quality directly affects HPM’s effectiveness and ability to continue stable operation.
	Capital investment	Gertler, et al.^18^, El Said and Stammer^20^, Wang^1^, Wang, et al.^43^	Capital investment is an important safeguard for HPM.
	Income situation	El Said and Stammer^20^, Shi, et al.^82^, Feng Li, et al.^83^	The income situation of highways affects the rational allocation of HPM resources.
	Mechanical efficiency	Ruiz Rodríguez, et al.^22^, Wheat^46^	Mechanical efficiency affects the effectiveness and economic efficiency of HPM.
Management cognition	Public cognition	Harvey^45^, Love, et al.^44^, Wheat^46^	The degree of public cognition directly affects the difficulty of HPM management.
	Personnel cognition	Al-Shabbani, et al.^47^, Zuluaga, et al.^48^	The degree of personal cognition directly affects their ability to drive safely and civilly on highways.
	Department cognition	Fei Guo and Zhang^49^, Zhao^50^, Sun^51^	The maintenance department has an in-depth understanding of the purpose and significance of HPM to accurately grasp and implement the maintenance measures.
	Manager capability	Greven, et al.^53^, Ying Liu, et al.^41^, Huo^54^, Greven, et al.^53^, Wang^84^	Having excellent managerial capabilities is the way to better promote HPM management.
	Sense of responsibility	Menges^52^, Huo^54^	The sense of responsibility of the maintenance staff guarantees that maintenance work is carried out properly.
Management decision	Management mode	Lee, et al.^3^, Mohamed, et al.^30^, Wang, et al.^43^, Wei^85^, Hou^86^	To meet the need for fast, efficient and high-quality decision-making under high-load maintenance operations, there is an urgent need to develop scientific and efficient management modes.
	Route selection	Borghetti, et al.^57^, Yu, et al.^60^, Wang, et al.^43^, Ji^87^	Scientific and reasonable route selection reduces the safety risk of highway operation.
	Timing determination	Kebede, et al.^56^, Yin, et al.^58^, Rodoplu, et al.^59^, You, et al.^62^, Guan, et al.^88^	Determine the optimal maintenance time point reduces the safety risk of highway operation.
	Measure effect	Zou, et al.^14^, Amarasiri and Muhunthan^16^, Wang, et al.^43^	To predict the benefits of maintenance efforts, implementation effects need to be measured.
	Maintenance cost	Lei, et al.^61^, Naseri, et al.^11^, You, et al.^62^, Li, et al.^12^, Liu, et al.^15^	Maintenance costs are directly related to the development, implementation and effectiveness of management decisions.
Management technology	New technology application	Kebede, et al.^56^, Kumar Gannina, et al.^63^, Yang, et al.^64^, Naseri, et al.^11^, Humayun, et al.^23^, Kruachottikul, et al.^66^	To address the current situation of heavy maintenance tasks, new technologies can be applied to improve the durability of pavements and extend the service life of highways.
	Personnel technology	Ruiz Rodríguez, et al.^22^, Tang^68^, Wang^69^	Improving personal technical skills helps to improve the effectiveness of maintenance management.
	Information utilization	Pan, et al.^19^, Kumar Gannina, et al.^63^, Lei, et al.^65^, Hijji, et al.^72^, Tezel and Aziz^73^	To develop effective maintenance programs, information needs to be fully explored and utilized.
	Information acquisition	Pan, et al.^19^, Kumar Gannina, et al.^63^, Zhang, et al.^70^, Jiang, et al.^71^, Hijji, et al.^72^	To fully understand the condition and problems of highways, adequate acquisition is needed.
External condition	Government attention	Gertler, et al.^18^, Yang, et al.^21^, Liu^74^, Hong Zhang, et al. ^5^	Government attention can increase investment and support for HPM management.
	Market competition	Yarmukhamedov, et al.^76^, Wu, et al. ^77^	Competition in the market helps the development of the highway maintenance industry.
	Work risk	Yao, et al.^78^, Sabatino, et al.^79^	The risky nature of maintenance work exacerbates the difficulty of maintenance work to some extent.
	Climate impact	Kebede, et al.^56^, Hernandez, et al.^80^, Sentic, et al.^81^, Liu, et al.^15^, Liu, et al.^17^	Maintenance work is often in the field, open air and other environments, vulnerable to climatic factors.

### Questionnaire survey

This paper builds on the literature review and draws on existing well-established scales based on the identified factors in Sect. 3.1, thus setting the measurement items. The questionnaire items were amended to form a questionnaire on HPM management factors through expert interviews and small sample testing. This study distributed the questionnaire through a combination of online and offline methods to collect survey data. Specifically, this was accomplished by sending questionnaire links to respondents through social media platforms such as WeChat and QQ and adopting a combination of self-administered questionnaires and face-to-face interviews for comprehensive data collection. The questionnaire was distributed to 300 respondents. A total of 260 valid responses were collected, resulting in a response rate of 86.7%. Invalid questionnaires were excluded on the basis of the following criteria: (1) questionnaires that left too many questions unanswered, (2) contradictory choices, and (3) almost identical to others.

The sample characteristics are shown in Table 2. Among them, 59.62% were male, and 40.38% were female. The majority of the survey respondents were under 45 years old (90.38%), had a bachelor’s degree (61.54%), and have been working for 6–15 years (53.85%). The main issuing units are the maintenance unit, detection unit, construction unit, advisory unit, and supervision unit. This observation indicates that respondents’ source compositions are consistent with reality and can reflect the actual situation to a certain extent.

**Table 2:** Characteristics of the sample.

Indicators	Item	Number	Percentage	Cumulative percentage
Gender	male	155	59.62	59.62
	female	105	40.38	100
Education background	specialty and below	100	38.46	38.46
	undergraduate	117	45.00	83.46
	master	30	11.54	95.00
	doctor	13	5.00	100.00
Age	<=25	78	30.00	30.00
	26–35	105	40.38	70.38
	36–45	52	20.00	90.38
	>=45	25	9.62	100.00
Working life	<=2	15	5.77	5.77
	3–5	60	23.08	28.85
	6–15	140	53.85	82.70
	>=15	45	17.30	100.00
Working unit	maintenance unit	78	30.00	30.00
	detection unit	36	13.85	43.85
	construction unit	84	32.31	76.16
	advisory unit	42	16.15	92.31
	supervision unit	20	7.69	100.00

### Exploratory factor analysis

To analyze the factors that influence HPM obtained from the questionnaire survey, the Kaiser‒Meyer‒Olkin (KMO) statistic method and Bartlett’s test of sphericity were used. From the data in Table 3 shows, the KMO value was obtained as 0.778, and the Bartlett’s spherical test chi-square value was 2426.642, with a significance less than 0.01. This finding indicates that the HPM scale is suitable for factor analysis.

**Table 3:** KMO and Bartlett’s test.

Kaiser‒Meyer‒Olkin Measure of Sampling Adequacy		0.778
Bartlett’s test of sphericity	Approx Chi-Square	2426.642
	Df	325
	Sig	0.000

To identify the most representative variables, variables with eigenvalues equal to or greater than 1.0 were selected as major factors. As presented in Table 4, a total of six major factors were chosen, with a cumulative variance contribution of 94.522%. The factor names were assigned after the component matrix was rotated, which yielded the following six factors: the management system, management resources, management cognition, management decisions, management technology, and external conditions. Only observed variables with factor loadings greater than 0.5 were included in the analysis, whereas those with loadings less than 0.5 were excluded. The final selection of 26 factors for HPM was based on group discussion and expert opinion, which excluded system feedback and a sense of responsibility. The validity of the factor analysis was strong, as confirmed by the high cumulative variance contribution of the six factors. Table 5 summarizes the 26 major HPM management factors.

**Table 4:** Total variance explained.

Component	Initial Eigenvalues			Rotation Sums of Squared Loadings		
	Total	Variance	Cumulative	Total	Variance	Cumulative
1	19.536	61.049	61.049	6.375	19.923	19.923
2	3.117	9.740	70.789	5.777	18.054	37.977
3	2.534	7.920	78.709	5.136	16.050	54.027
4	2.109	6.590	85.299	5.095	15.921	69.947
5	1.796	5.612	90.911	4.932	15.414	85.361
6	1.155	3.611	94.522	2.932	9.161	94.522
7	0.735	2.297	96.819			

**Table 5:** Major HPM management factor.

Dimension	Factor	Factor loading
Management system	System establishment	0.930
	System perfection	0.851
	System execution	0.834
Management resource	Machinery allocation `	0.945
	Material supply	0.835
	Material quality	0.780
	Capital investment	0.784
	Income situation	0.764
	Mechanical efficiency	0.796
Management cognition	Personnel cognition	0.788
	Public cognition	0.917
	Department cognition	0.813
	Manager capability	0.818
Management decision	Management mode	0.946
	Route selection	0.797
	Timing determination	0.750
	Measure effect	0.788
	Maintenance cost	0.806
Management technology	New technology application	0.878
	Personnel technology	0.813
	Information utilization	0.813
	Information acquisition	0.525
External condition	Government attention	0.944
	Market competition	0.792
	Work risk	0.801
	Climate impact	0.802

## Degree of importance of major HPM management factors

To distinguish the degree of importance of management factors, this paper uses the SNA method to explore these factors in depth to select key, hub and non-key factors. The research invited ten experts to score the relationships between factors, with a score of 1 indicating influential factors and 0 indicating noninfluential factors. The resulting relationship matrix of factors was then constructed and imported into UCINET 6 software, to calculate the relative degree of centrality, as shown in Table 6. Information acquisition, system perfection, system establishment, government attention, new technology application, capital investment, and the management mode are also important and are defined as key factors that are the top considerations for enhancing management effectiveness. System execution, manager capability, personnel technology, maintenance cost, information utilization, income situation, measure effect, public cognition, department competition, and personnel cognition connect key factors and non-key factors, which are defined as hub factors. Route selection, machinery allocation, timing determination, market competition, material supply, work risk, mechanical efficiency, material quality, and climate impact are at the edge, which are defined as non-key factors.

**Table 6:** Measurement analysis results.

Factor	Out-degree	In-degree	C_IRD_(X)	Rank
information acquisition	14.00	25.00	0.78	1
system perfection	13.00	20.00	0.66	2
system establishment	12.00	21.00	0.63	3
government attention	17.00	14.00	0.62	4
new technology application	15.00	15.00	0.60	5
capital investment	14.00	16.00	0.60	6
management mode	15.00	14.00	0.58	7
system execution	14.00	14.00	0.56	8
manager capability	18.00	10.00	0.56	9
personnel technology	14.00	14.00	0.56	10
maintenance cost	15.00	13.00	0.56	11
information utilization	13.00	11.00	0.48	12
income situation	14.00	10.00	0.48	13
measure effect	11.00	13.00	0.48	14
public cognition	10.00	13.00	0.46	15
department competition	14.00	9.00	0.46	16
personnel cognition	10.00	11.00	0.42	17
route selection	12.00	7.00	0.38	18
machinery allocation	4.00	13.00	0.34	19
timing determination	11.00	6.00	0.34	20
market competition	10.00	5.00	0.32	21
material supply	7.00	8.00	0.30	22
work risk	8.00	6.00	0.28	23
mechanical efficiency	4.00	9.00	0.26	24
material quality	6.00	7.00	0.26	25
climate impact	9.00	0.00	0.18	26

**Figure 2:**
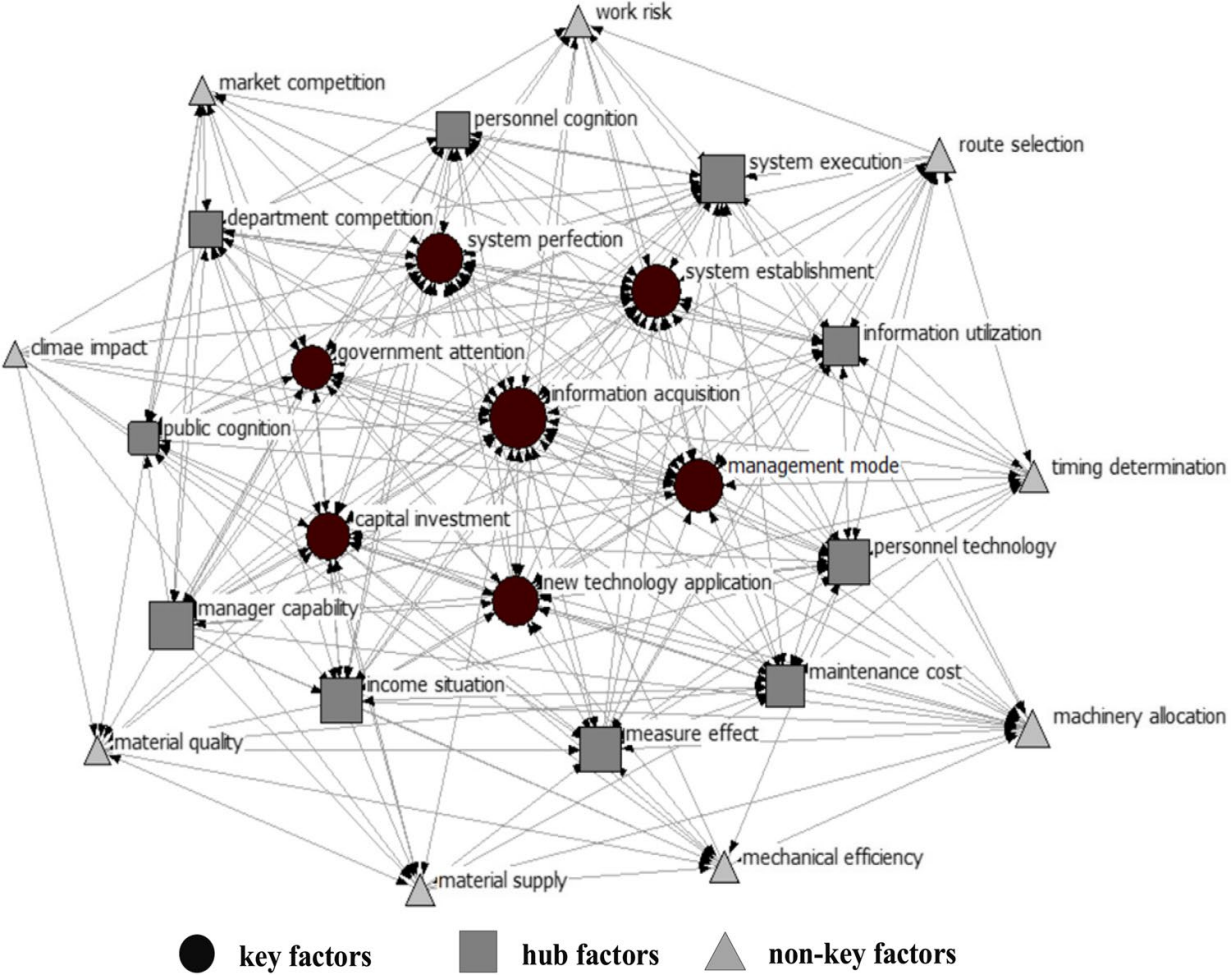
Network relation graph.

Netdraw is used to draw the relationship graph of the major HPM management factors, as shown in Fig. 2. The graph highlights the critical role of information acquisition in HPM management, as evidenced by its highest relative centrality and its ability to radiate to other areas. To ensure effective preventive maintenance management, managers must possess comprehensive knowledge of systems, technologies, funds, materials, and external dynamics, enabling them to make informed decisions^89^. Highways have the attributes of high traffic volume, perfect equipment, and high technological content. However, the existing maintenance management system has defects such as insufficient theoretical innovation and vague objectives, which cannot achieve the expected effect of preventive maintenance management^90^. Additionally, the continuous evolution of maintenance technology, the emergence of new materials, and the steady progress of equipment, coupled with substantial capital investment, are the primary drivers of enhanced technology, materials, and equipment^91^. From the above discussion, it is clear that the relative degree of centrality of key factors such as information acquisition and system perfection is greater. System execution, managerial capability, etc., as the “link” between key and non-key factors, have a greater impact on both but less of an effect on the effectiveness of HPM management. Therefore, hub factors are relatively less centralized. With the cooperation of the government and the units, non-key factors such as route selection and machinery allocation have little impact on management efficiency, which means the lowest level of centralization.

## Pattern of influence of major HPM management factors

### Causal-loop diagram

**Figure 3:**
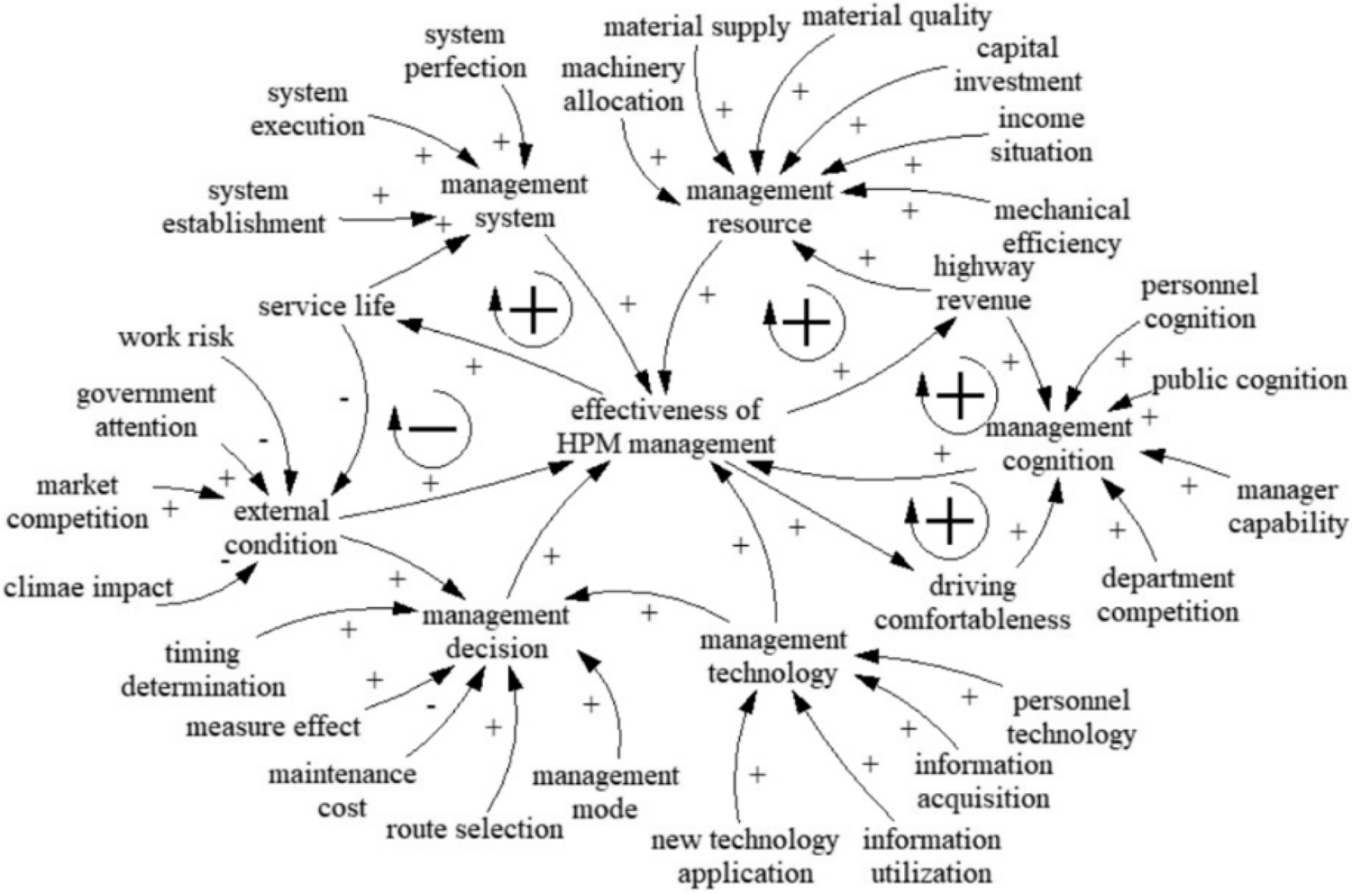
Causal-loop diagram.

On the basis of the relationships among the major HPM management factors, a causal loop diagram between management factors and the effectiveness of HPM management can be built to describe the interaction relationships, as shown in Fig. 3. There are 5 relationship loops, which consist of 4 positive relationship loops and 1 negative relationship loop. The dynamic interaction behavior of the variables in each relationship loop is interpreted below.

1. Loop 1: Management cognition → +effectiveness of HPM management → +driving comfortableness → +management cognition.

Loop 1 is a positive feedback loop. Improving management cognition enhances managers’ positive severity of highway management and simulates the implementation of preventive maintenance, promoting the effectiveness of HPM management. In this situation, the conditions and quality of highways can be well maintained, which enables drivers to feel more comfortable when driving. Thus, management perceptions are enhanced.

(2) Loop 2: Management cognition → +effectiveness of HPM management → +highway revenue → +management cognition.

Loop 2 is a positive feedback loop. The state vigorously promotes the HPM concept and increases conservation implementation. This prolongs the service of the road and further improve the effectiveness of HPM management. Therefore, highway revenue increases as more drivers choose highways. In this way, improved revenue stimulates managers to further improve their management cognition.

(3) Loop 3: Management resources → +effectiveness of HPM management → +highway revenue → +management resources.

Loop 3 is a positive feedback loop. The effectiveness of HPM management can be significantly improved by vigorously developing management resources and rationally allocating resources. In turn, the revenue of highways is on an upward trend, thus improving the supply of management resources and mechanical efficiency.

(4) Loop 4: Management system → +effectiveness of HPM management → +service life → +management system.

Loop 4 is a positive feedback loop. The state improved the management system and realized the transformation of information, automation and intelligence of maintenance management, which contributes to promoting the effectiveness of HPM management. This significantly extends the service life of highways. In this situation, managers are motivated to further improve the management system.

(5) Loop 5: External condition → +effectiveness of HPM management → +service life → -external condition.

Loop 5 is a negative feedback loop. The quality of preventive maintenance can be affected by external conditions, including advanced technology, advanced equipment, quality materials, strict management, and especially the appropriate season. Therefore, the lower the fluctuation of external conditions, the greater the effectiveness of HPM management, and the longer the service life of the highway. However, as the service life increases, the external conditions worsen.

### Stock–flow diagram

**Figure 4:**
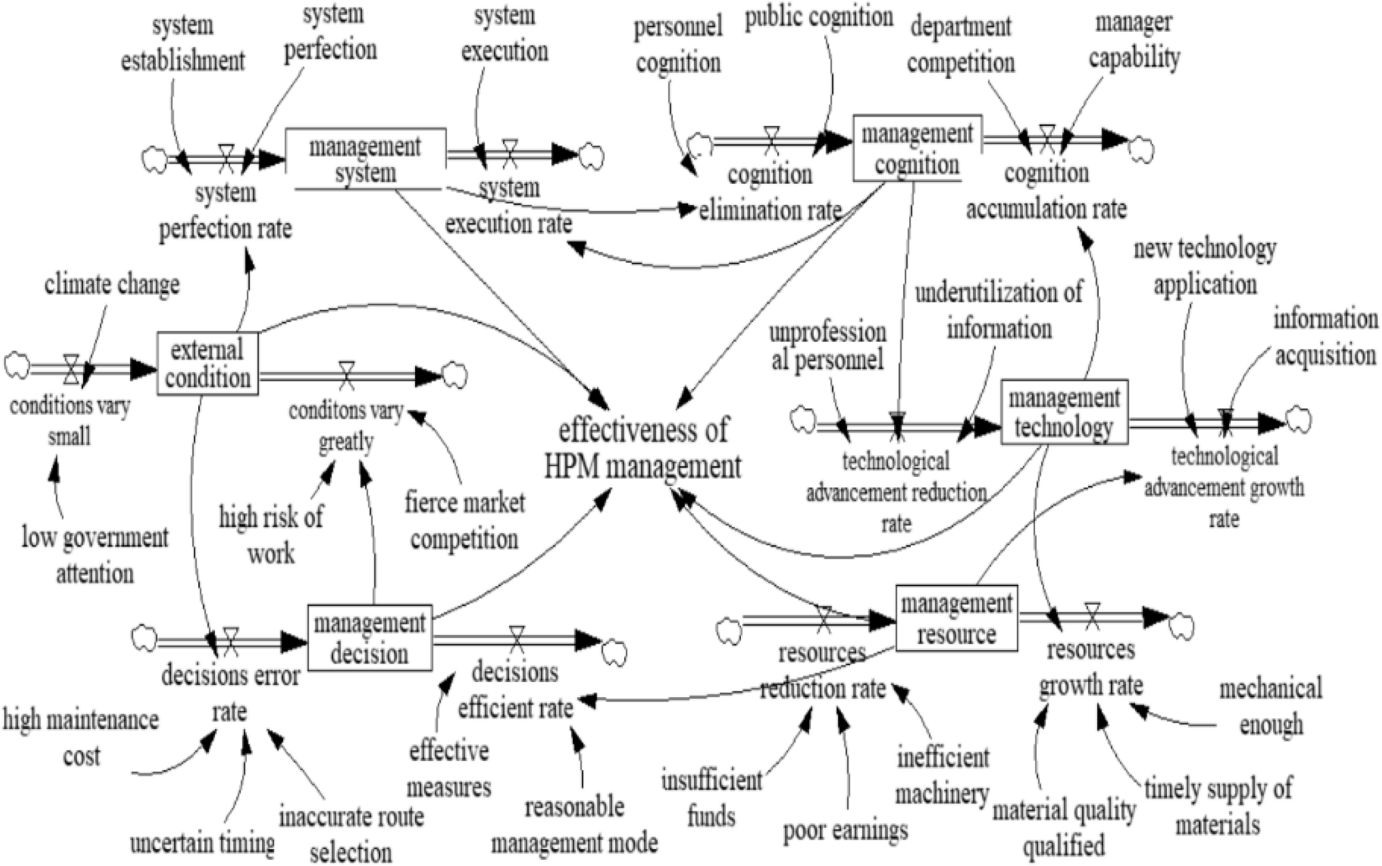
Stock-flow diagram.

After the main factors and their interactions involved in the whole system are identified, a stock‒flow diagram using Vensim software is developed so that the SD model can be run to simulate the internal dynamic relationships between the factors, as shown in Fig. 4.

### State transition equation

As an essential part of the SD model, the state transition equation describes the dynamic change patterns of the factors in the model, which provides theoretical support for simulation. The SD model for the major HPM management factors involves the following state transition equations, shown in Table 7.

**Table 7:** State transition equations table.

Variable Category	Name	Equation
Horizontal variables	management system	INTEG (management system change volume, 0)
	management resource	INTEG (management resource change volume, 0)
	management cognition	INTEG (management cognition change volume, 0)
	management decision	INTEG (management decision change volume, 0)
	management technology	INTEG (management technology change volume, 0)
	external condition	INTEG (external condition change volume, 0)
Rate variables	management system change volume	system execution rate-system perfection rate
	management resource change volume	resources growth rate-resources reduction rate
	management cognition change volume	cognition accumulation rate-cognition elimination rate
	management decision change volume	decisions efficient rate-decisions error rate
	management technology change volume	technological advancement growth rate-technological advancement reduction rate
	external condition change volume	conditions vary greatly conditions vary small
Auxiliary variables	effectiveness of HPM management	w_1_×management system + w_2_×management resource + w_3_×management cognition + w_4_×management decision + w_5_×management technology + w_6_×external condition
	system perfection rate	a_1_×system establishment + a_2_×system perfection+a_3_×external condition
	system execution rate	b_1_×system execution + b_2_×management cognition
	resources reduction rate	c_1_×insufficient funds + c_2_×poor earnings+ c_3_ ×inefficient machinery
	resources growth rate	d_1_×material quality qualified + d_2_×timely supply of materials + d_3_×mechanical enough + d_4_×management technology
	cognition elimination rate	e_1_×personnel cognition + e_2_×public cognition+ e_3_×management system
	cognition accumulation rate	f_1_×department competition + f_2_×manager capability+ f_3_×management technology
	decisions error rate	g_1_×high maintenance cost + g_2_×uncertain timing+ g_3_×inaccurate route selection + g_4_×external condition
	decisions efficient rate	h_1_×effective measures + h_2_×reasonable management mode + h_3_×management resource
	Technological advancement reduction rate	k_1_×unprofessional personnel + k_2_×underutilization of information + k_3_×management cognition
	technological advancement growth rate	m_1_×new technology application + m_2_×information acquisition + m_3_×management resource
	conditions vary small	o_1_×climate change + o_2_×low government attention
	conditions vary great	p_1_×high risk of work + p_2_×fierce market competition+ p_3_×management decision

Note: w_1_, w_2_, w_3_, w_4_, w_5_, w_6_, a_1_, a_2_, a_3_, b_1_, b_2_, c_1_, c_2_, c_3_, d_1_, d_2_, d_3_, e_1_, e_2_, e_3_, f_1_, f_2_, f_3_, g_1_, g_2_, g_3_, h_1_, h_2_, h_3_, h_4_, k_1_, k_1_, k_1_, k_1_, m_1_, m_1_, m_1_, o_1_, o_1_, p_1_, p_1_, p_1_ are parameters and satisfy ∑a,∑b,∑c,∑d,∑e,∑f,∑g,∑h,∑k,∑m,∑o,∑p are 1.

By setting the initial value of major factors as the mean of secondary factors, it can be ensured to a certain extent that the initial state of major factors can better synthesize the impact of these secondary factors. Further, the initial values of the secondary factors are set as the means of the out-degree and in-degree, which are determined by ten HPM-related experts combining professional knowledge and practical experience. Finally, the parameters of secondary factors can be modified and determined by standardized relative centrality, which can eliminate the barriers to comparison between networks of different sizes. Overall, this means that the modification and determination of parameters is more impartial and reasonable, making the model more accurate and closer to reality.

According to Table 6; Fig. 2, the sums of the relative degrees of centrality in the management system, management resources, management cognition, management decisions, management technology and external conditions are 1.85, 2.24, 1.9, 2.34, 2.42, and 1.4, respectively. The sum of the relative degree centers of all secondary factors is 12.15, so w_1_ = 0.15, w_2_ = 0.19, w_3_ = 0.16, w_4_ = 0.19, w_5_ = 0.2, and w_6_ = 0.11. Following the above principles, the parameter results are shown in Table 8.

**Table 8:** Equation parameters.

Parameter	Value	Parameter	Value
management system	3.1	public cognition	2.3
management resource	1.87	department competition	2.3
management cognition	2.37	manager capability	2.8
management decision	2.34	high maintenance cost	2.8
management technology	3.03	uncertain timing	1.7
external condition	1.72	inaccurate route selection	1.9
system establishment	3.1	effective measures	2.4
system perfection	3.3	reasonable management mode	2.9
system execution	2.8	unprofessional personnel	2.8
insufficient funds	3	underutilization of information	2.4
poor earnings	2.4	new technology application	3
inefficient machinery	1.3	information acquisition	3.9
material quality qualified	1.3	climate change	0.9
timely supply of materials	1.5	low government attention	3.1
mechanical enough	1.7	high risk of work	1.4
personnel cognition	2.1	fierce market competition	1.5

w_1_ = 0.15,w_2_ = 0.19,w_3_ = 0.16,w_4_ = 0.19,w_5_ = 0.2,w_6_ = 0.11,a_1_ = 0.34,a_2_ = 0.36,a_3_ = 0.3,b_1_ = 0.3,b_2_ = 0.7,c_1_ = 0.45,c_2_ = 0.35,c_3_ = 0.2,d_1_ = 0.12,d_2_ = 0.13,d_3_ = 0.15,d_4_ = 0.60,e_1_ = 0.22,e_2_ = 0.24,e_3_ = 0.54,f_1_ = 0.24,f_2_ = 0.3,f_3_ = 0.46,g_1_ = 0.24,g_2_ = 0.15,g_3_ = 0.17,g_4_ = 0.44,h_1_ = 0.19,h_2_ = 0.25,h_3_ = 0.56,k_1_ = 0.23,k_2_ = 0.2,k_3_ = 0.57,m_1_ = 0.25,m_2_ = 0.32,m_3_ = 0.43,o_1_ = 0.23,o_2_ = 0.77,p_1_ = 0,2,p_2_ = 0.23,p_3_ = 0.54.

### Simulation results and analysis

**Figure 5:**
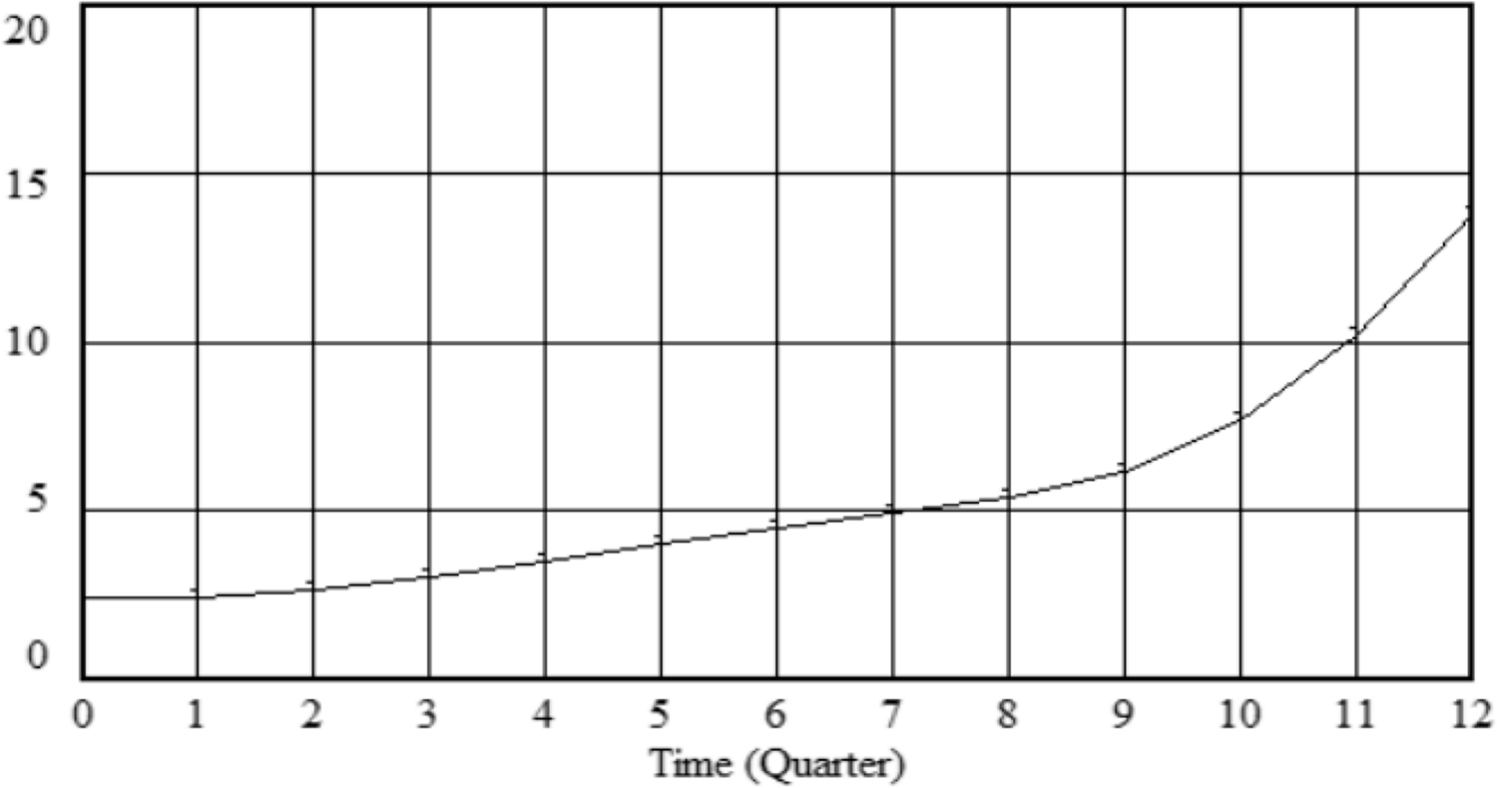
Evolution simulation effectiveness of HPM management.

**Figure 6:**
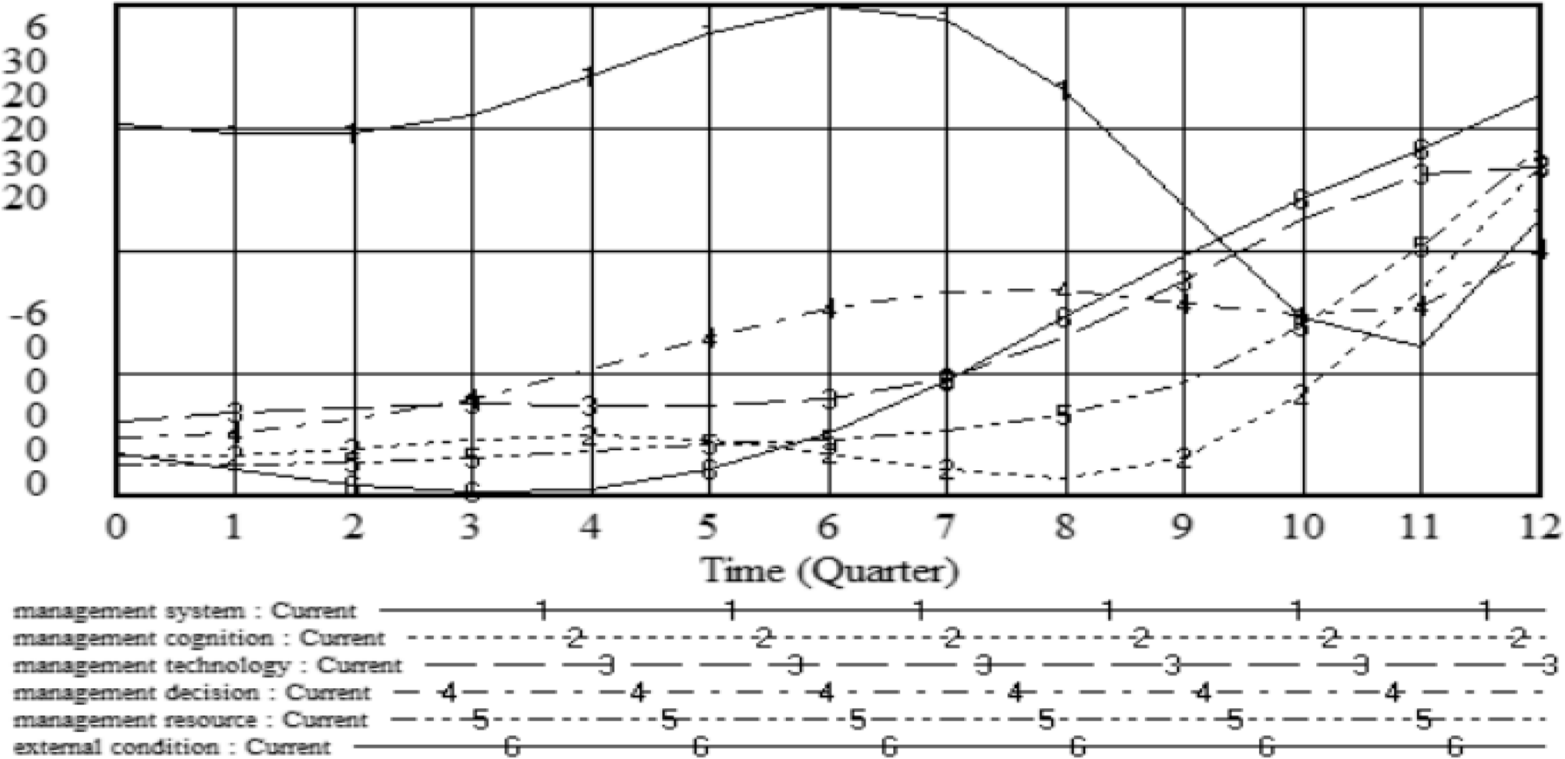
Evolutionary simulation of major HPM management factors.

This research uses Vensim software to simulate the pattern of influence of management effectiveness, setting INITIAL TIME = 0, FINAL TIME = 12, TIME STEPT = 1, and UNTIS for the TIME as the Quarter. The simulation results are shown in Figs. 5 and 6. In recent years, paying equal attention to construction and maintenance has become the industry orientation for the development of technology in the field of highway maintenance. With the active cooperation and participation of the state, government, and various units, management effectiveness has continued to improve. Thus, the changes in the effectiveness of HPM management show a gradual upward trend, which is consistent with the actual situation.

1. The effect of the change of the rule in the management system on management effectiveness.

There is a slight decrease in the initial stage, a gradual increase in the middle stage, a marked decline in the middle and later stages and a rapid increase in the later stage. This can be explained by the inadequate management system and weak execution in the early stage. Moreover, it is not sufficient to mine the data of the management system and apply it to maintenance decisions. In the middle stage, the management system gradually becomes applicable through continuous learning and innovation, which can improve the effectiveness of HPM management. Over time, the management system meets the criteria and reaches a state of saturation. Hence, the effect rule is significantly reduced compared with that in the middle stage. In the later stage, management effectiveness is strengthened by improving the management system and formulating a reasonable HPM postevaluation mechanism.

(2) The effect of changes in management resources and management technology on management effectiveness.

It shows a slowly increasing trend. As the state actively organizes various preventive maintenance seminars, it encourages relevant personnel to learn advanced technology about preventive maintenance and provides funds for special preventive maintenance and standardized research on maintenance materials. Thus, it gradually forms maintenance materials and technologies with independent intellectual property rights. Advanced technology, new materials, and sufficient capital can provide infinite possibilities to improve the management effect.

(3) The effect of changes in management cognition on management effectiveness.

There is no significant change in the early stage, but it decreases in the middle stage and increases in the later stage. This can be explained by the fact that there are still some maintenance personnel with conservative ideas even though the state actively promotes HPM management. In addition, their professional qualities are mixed. For example, they are relatively old and do not understand the economic benefits of preventive maintenance. Therefore, the impact of management cognition is relatively small in the early stage and decreases in the middle stage. With the increasingly prominent benefits of preventive maintenance, its concept and mode have been widely recognized. In addition, the concept of HPM management is popularized by summarizing the experiences of pilot cities across the country. Moreover, the development of integrated equipment, onsite condition control and appropriate contract management has promoted the healthy development of preventive maintenance. Furthermore, the maintenance management department has gradually formed a relatively intelligent HPM management system, thus improving management effectiveness.

(4) The effect of changes in management decisions on management effectiveness.

Management effectiveness increases in the early stage, decreases slowly in the middle stage, and increases in the later stage. This is because the rapid development of information, data, and intelligence determines the scientific nature of maintenance time. Moreover, carrying out preventive maintenance in time can promote management effectiveness. In the middle stage, adverse effects appear due to high maintenance costs, a shortage of funds, and backward management modes. In the later stage, with the development of high-speed detection technology for pavement performance and the establishment of a digital management platform, the scientific and intelligent level of maintenance management has improved. Thus, the effectiveness of HPM management is promoted.

(5) The effect of changes in external conditions on management effectiveness.

There is an initial decrease and a slow increase after the middle stage. This is due to the relatively weak maintenance technology investment mechanism and market operation mechanism in the early stage, which constrains the improvement of management effectiveness. In the middle stage, the concept of preventive maintenance is widely accepted, leading to market competition and the rationality of pavement structure design. The interaction of great attention and information ensures the safety of maintenance personnel, increases the frequency of pavement inspections, and promotes effective management.

## Discussions

### Implications

In this paper, a hybrid EFA-SNA-SD approach is used to integrate the major management factors of HPM systematically, distinguish their degree of importance, and analyze their patterns of influence. The findings enriched and broadened the development of preventive maintenance concepts, contributing to the formation of a sustainable transportation system.

The study identified 26 major HPM management factors, which were categorized into six areas. This has improved our ability to interpret maintenance problems and enables effective action to be taken in response to HPM management issues. By integrating these factors into preventive maintenance management, the overall quality of highway maintenance can be improved, create a safe and efficient traffic system, and meet future high-demand, high-efficiency, and high-quality highway services for sustainable development.

The study revealed that the major HPM management factors were hierarchical and mutually constraining. Therefore, this paper distinguished them into key, hub, and non-key factors. This facilitated preventive maintenance work and provided a direction for the efficient improvement of preventive maintenance management effectiveness. The key management factors in daily HPM management include a suitable management mode, adequate preparation, and reasonable financial support. However, limitations in maintenance technology, equipment, information, and changing social industry environments can limit the choice of the best management model, the speed of obtaining the best maintenance information, and the reasonable allocation of funds. Therefore, appropriate maintenance plans need to be developed to achieve sustainable development of highways. Multilevel implementation measures are key to improving the effectiveness of HPM management. By constructing the SD model, this study revealed that the pattern of influence of different management factors on the effectiveness of HPM management varied and generally showed an increasing trend. On the basis of these findings, the following suggestions are proposed. First, we should follow the principle of adapting measures to local conditions and ensuring consistency between power and responsibility to gradually improve the HPM management system, especially the strengthening of the operation mechanism. Further, the government should increase the investment in preventive maintenance funds and allocate them reasonably while also providing regular technical guidance to maintenance personnel in improving their professional ability. Furthermore, the concept and long-term benefits of preventive maintenance management should be promoted actively and the correct awareness of preventive maintenance management must be established. Finally, we recommend collecting and integrating highway data and using GIS to improve the information and intelligence of HPM. These measures provide strong theoretical support and technical guarantees for the implementation of preventive maintenance management.

### Limitations and recommendations

In this paper, a hybrid approach of EFA-SNA-SD is presented to integrate and analyze the major management factors of the effective HPM. However, there are several limitations in the research process that need to be addressed.

First, as the development of HPM management continues, the major management factors may change over time. Therefore, future research can focus on the development trend of preventive maintenance and adjust the management factors accordingly. Further, the data is limited, and the sample size is not comprehensive enough to represent all regions. Future research could benefit from expanding the sample size and collecting data from different regions. Establishing long-term performance observation stations for pavement performance across the country to provide a more comprehensive and accurate scientific basis for preventive maintenance decisions is suggested. Finally, it is acknowledged that the methodology can be further improved. Considering programming software such as R language and MATLAB for clustering and visual analysis of management factors is recommended, which aids in achieving a combination of computer technology and the integration of HPM management concepts.

Overall, despite these limitations, this study contributes to the understanding of HPM management and provides theoretical and practical references for enhancing the benefits of preventive maintenance management to form a sustainable transportation system. It is believed that further research in this area can lead to significant improvements in the effectiveness of HPM management.

## Conclusions

In conclusion, this research aimed to identify the major HPM management factors and their dynamic effects on the effectiveness of HPM management via a hybrid EFA-SNA-SD approach. The research identified 26 major HPM management factors that are categorized into six dimensions: the management system, management resources, management cognition, management decisions, management technology, and external conditions. Information acquisition, system perfection, system planning, etc., are identified as key factors critical to the effectiveness of HPM management. System execution, manager capability, organizational support, etc., are identified as hub factors that significantly influence HPM management effectiveness. Route selection, machinery allocation, pavement structure, etc., are identified as non-key factors that have less impact on the effectiveness of HPM management. The SD model developed in this study demonstrates that different management factors have varying effects on the effectiveness of HPM management. The results indicate that effective management strategies require a holistic approach that considers all dimensions of HPM management. Furthermore, the model shows that the effectiveness of HPM management can be improved through continuous monitoring and adjustment of management factors.

The findings of this research have significant implications for the sustainable development of highways. The results can guide policy-makers and highway managers in developing effective HPM management strategies that enhance the durability, safety, and cleaner production of highway infrastructure, thus contributing to a more sustainable transportation system.

## Electronic supplementary material

Below is the link to the electronic supplementary material.

Supplementary Material 1

## Data Availability

Data is provided within the manuscript or supplementary information files.
